# A Signaling Pathway Involving TGF-β2 and Snail in Hair Follicle Morphogenesis

**DOI:** 10.1371/journal.pbio.0030011

**Published:** 2004-12-28

**Authors:** Colin Jamora, Pedro Lee, Pawel Kocieniewski, Mohamad Azhar, Ryoichi Hosokawa, Yang Chai, Elaine Fuchs

**Affiliations:** **1**Howard Hughes Medical Institute, Laboratory of Mammalian Cell Biology and DevelopmentThe Rockefeller University, New York, New YorkUnited States of America; **2**Department of Molecular Genetics, Biochemistryand Molecular Biology, University of Cincinnati, CincinnatiUnited States of America; **3**Center for Craniofacial Molecular Biology, University of Southern CaliforniaLos Angeles, CaliforniaUnited States of America; Duke University Medical CenterUnited States of America

## Abstract

In a common theme of organogenesis, certain cells within a multipotent epithelial sheet exchange signals with their neighbors and develop into a bud structure. Using hair bud morphogenesis as a paradigm, we employed mutant mouse models and cultured keratinocytes to dissect the contributions of multiple extracellular cues in orchestrating adhesion dynamics and proliferation to shape the cluster of cells involved. We found that transforming growth factor β2 signaling is necessary to transiently induce the transcription factor Snail and activate the Ras-mitogen-activated protein kinase (MAPK) pathway in the bud. In the epidermis, *Snail* misexpression leads to hyperproliferation and a reduction in intercellular adhesion. When *E-cadherin* is transcriptionally down-regulated, associated adhesion proteins with dual functions in signaling are released from cell-cell contacts, a process which we demonstrate leads to Ras-MAPK activation. These studies provide insights into how multipotent cells within a sheet are stimulated to undergo transcriptional changes that result in proliferation, junctional remodeling, and bud formation. This novel signaling pathway further weaves together the web of different morphogens and downstream transcriptional events that guide hair bud formation within the developing skin.

## Abstract 2

The study of hair follicle morphogenesis provides insights into how cells within a sheet can be triggered to proliferate, remodel, and form buds - a recurring theme in development

## Introduction

Mammalian development involves the morphogenesis of complex three-dimensional structures from seemingly uniform sheets or masses of cells. A simple bud-like structure initiates the formation of many organs, including lungs, spinal cord, mammary glands, and hair follicles [1]. The multipotent, adhering epithelial cells are typically attached to an underlying basal lamina that polarizes the epithelial sheet and separates it from surrounding mesenchyme. Budding morphogenesis is guided by a reciprocal exchange of signals between epithelium and mesenchyme to specify the identity of the organ that will form and to govern its growth.

At the helm of these molecular communication pathways are Wnts, bone morphogenic proteins (BMPs), transforming growth factor βs (TGF-βs), and fibroblast growth factors (FGFs). Through activation of cell surface transmembrane receptors, these external signaling molecules trigger distinct cascades of intracellular events that culminate in changes in gene expression, growth, and differentiation [2]. How this constellation of signals collaborates in tailoring each budding process so that it executes a distinct morphogenetic program has yet to be comprehensively defined. However, the process appears to be patterned at the initial stages of bud formation, since the relative importance of these pathways and their downstream effectors differ as buds begin to develop and cell fates are specified.

The development of a bud requires a number of coordinated changes in the behavior of the targeted cells within an epithelial sheet. The process must be accompanied by alterations in the proliferation, polarity, shape, and adhesiveness of selected cells, as well as by modifications in their underlying basal lamina. Thus, extracellular epithelial-mesenchymal crosstalk must be intricately orchestrated to couple the determination of distinct cell fates with the contemporaneous remodeling of the physical and structural properties of the cell.

Among the few dispensable organs, hair follicles offer an excellent model system to study epithelial bud formation. Mammalian skin epithelium begins as a single sheet of multipotent ectodermal cells. During development, specialized mesenchymal cells populate the skin in a spatially defined pattern to initiate the complex epithelial-mesenchymal crosstalk that will specify the bud [3]. Once committed, a small cluster of epithelial cells, the placode, instructs a group of underlying mesenchymal cells to condense and form the nascent dermal papilla, which will be a permanent fixture of the hair follicle. Subsequent exchanges between the placode and nascent dermal papilla result in further growth of the follicle into the underlying dermis, or down-growth, and eventual differentiation into the six concentric layers of the mature follicle.

Previously, we delineated how two respective epithelial and mesenchymal signals, Wnts and the BMP-inhibitory factor noggin, function in concert to induce lymphoid enhancer factor-1/β-catenin (LEF-1/β-catenin)-mediated gene transcription within the follicle placode [4]. The downstream changes elicited through convergence of these two early signaling pathways include down-regulation of the gene encoding E-cadherin, the prototypical epithelial cadherin that forms the transmembrane core of intercellular adherens junctions (AJs) [5]. We subsequently showed that when E-cadherin is transgenically elevated in mouse skin, hair follicle morphogenesis is blocked, suggesting that E-cadherin down-regulation is a critical event in governing the adhesion dynamics necessary for budding morphogenesis [4]. Like LEF-1, E-cadherin also binds to β-catenin. At sites of cell-cell contact, however, E-cadherin-β-catenin complexes recruit α-catenin, which in turn coordinates the associated actin polymerization dynamics necessary to stabilize nascent AJs and integrate the cytoskeleton across an epithelial sheet [6,7,8]. α-Catenin also binds to the class III Lin-1, Isl-1, Mec-3 (LIM) protein Ajuba (a member of the zyxin family of proteins), which appears to function dually in both adhesion and in activation of the Ras-mitogen-activated protein kinase (MAPK) pathway [9,10]. Through these links, AJs appear able to couple adhesion with cytoskeletal dynamics as well as with nuclear and cytoplasmic signaling. This provides a framework for conceptualizing why E-cadherin levels appear to impact upon a plethora of developmental processes (reviewed in [11]).

As we probed more deeply into the underlying mechanisms governing *E-cadherin* promoter activity, we were intrigued by the close proximity of the LEF-1/β-catenin binding site to a site known to bind the Snail/Slug family of zinc finger transcriptional repressor proteins [12,13,14,15]. Both activity of Snail and down-regulation of E-cadherin play pivotal roles in epithelial to mesenchymal transitions (EMTs), typified by the transformation of polarized, adhering epithelial cells into motile mesenchymal cells [16,17]. Bud formation differs from an EMT in that E-cadherin activity needs to be down-regulated but not prevented, so that adhesive junctions are remodeled rather than quantitatively impaired. Supportive of an underlying ability to fine-tune cadherin expression at the transcriptional level, Snail seems to have an additive effect with LEF-1/β-catenin in negatively modulating *E-cadherin* promoter activity [4].

In the present study, we discovered that Snail is expressed briefly at an early stage of hair bud formation, when E-cadherin down-regulation and activation of proliferation take place. Thereafter, Snail disappears and remains absent during subsequent follicle down-growth and maturation. This exquisite pattern appears to be functionally relevant since altering it in vivo correspondingly affects features associated with hair bud formation, including down-regulation of E-cadherin, increased proliferation, and repressed terminal differentiation. Although the temporal spike of Snail in the hair bud is reflected at the mRNA level and seems to follow Wnt signaling and BMP inhibition, LEF-1/β-catenin activation does not appear to induce *Snail* gene expression in embryonic skin keratinocytes. In contrast, we provide in vitro, transgenic (Tg), and gene targeting evidence to show that TGF-β2 and small phenotype– and mothers against decapentaplegic–related protein 2 (SMAD2) signaling are upstream inducers of *Snail* gene expression in skin epithelium. In the absence of TGF-β2 signaling and *Snail* gene expression, hair placodes can form, but further follicle down-growth is blocked. Our studies point to the view that Snail likely functions downstream of cell fate specification, at a stage where the bud begins to exhibit enhanced proliferation and migration.

## Results

### *Snail* mRNA and Protein Are Expressed Transiently at the Hair Bud Stage of Follicle Morphogenesis

Although Snail family members are most frequently associated with EMTs, they also participate in many malignant processes involving a down-regulation but not a quantitative abrogation of intercellular junctions [18]. The range of developmental processes in which Snail family members have been implicated thus includes the type of epithelial remodeling that is observed in hair follicle bud formation. Given our prior observation that exogenously added Snail can participate with LEF-1/β-catenin in down-regulating *E-cadherin* expression in keratinocytes [4], coupled with the established requirement for LEF-1/β-catenin in hair follicle morphogenesis [4,19], we turned to addressing whether Snail/Slug family members might also participate in the process.

**Figure 1:**
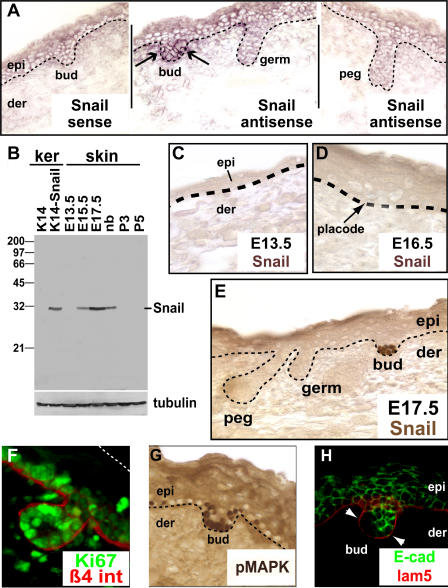
Snail Is Expressed Exclusively in the Hair Bud during Morphogenesis Embryos were either frozen in OCT embedding compound (A, F, and H) or embedded in paraffin (C, D, E, and G), and then sectioned (8 μm). (A) In situ hybridizations with *Snail* sense or antisense cRNA probes. Black dotted lines demarcate the basement membrane that separates the epidermis (epi) from dermis (der). Arrows point to *Snail* RNA expression, restricted to the hair bud stage of follicle morphogenesis. It was not seen in later hair germ or peg stages. (B) Expression of Snail protein coincides with hair development. Protein extracts were prepared from keratinocytes transfected with empty expression vector (K14), containing the K14 promoter or with the vector driving HA-tagged *Snail* (K14-*Snail*); or from whole skin from E13.5 to P5 animals, including newborn (nb). Equal amounts of proteins were then resolved by SDS-PAGE through 12% gels and subjected to Western blotting using either an affinity-purified Snail polyclonal antiserum, which we generated, or anti-tubulin (loading control). (C–E) Immunohistochemistry shows expression of Snail protein in the nuclei of cells within the hair and skin. (C) E13.5 skin with a single layered epidermis (epi) shows no Snail expression. (D) The first morphological sign that cells have adopted a hair follicle fate is a cluster of cells called a placode in E16.5 skin. Snail is not expressed at this stage of development. (E) Snail is expressed in the hair bud of E17.5 skin but not in later stages of development such as the germ or peg. (F) Immunofluorescence with anti-Ki67 (green) identifies the proliferating cells of the skin, restricted to the basal layer of the epidermis and developing hair follicles. Anti-β4 int labeling reveals the presence of the hemidesmosomal integrin β4, restricted to the base of cells adhering to the underlying basement membrane. The white dotted line marks the outermost surface of the skin. (G) Immunohistochemistry with pMAPK marks a subset of proliferating cells within the epidermis and hair bud. Anti-pMAPK labeling was consistently robust within the hair bud. (H) Immunofluorescence with anti-laminin 5 (lam5), which demarcates the basement membrane, and anti-E-cadherin (E-cad), a component of AJs. At the leading edge of the growing bud, cell-cell borders show markedly diminished anti-E-cadherin labeling (arrowheads).

PCR analyses identified transient *Snail* mRNA expression during a period of skin embryogenesis when waves of hair follicles are forming (unpublished data).To pinpoint specifically where *Snail* mRNA is expressed in the developing skin, we conducted in situ hybridization using a cRNA probe unique to the *Snail* 3′ untranslated region (UTR). Embryonic day 17.5 (E17.5) was chosen, since the multiple waves of follicle morphogenesis occurring at this time enabled us to evaluate Snail expression at different stages of the process. As shown in Figure 1A, specific hybridization was detected within the epithelium of nascent hair buds. By contrast, as follicles progressed further through their development (e.g., germ and peg stages), they exhibited no signs of hybridization (Figure 1A). The transient nature of *Snail* mRNA expression during follicle development was most apparent in hybridized skin sections containing follicles from two different waves of morphogenesis (as shown in Figure 1). Hybridizing hair buds from a later wave appeared juxtaposed with nonhybridizing follicles from an earlier wave.

To determine whether this transient nature of *Snail* mRNA expression is reflected at the protein level, we generated an antibody against the N-terminal sequence that resides upstream of the more conserved zinc finger domains. As judged by Western blot analysis, the antibody did not detect endogenous proteins from cultured keratinocytes, but it did yield a band of the expected size from keratinocytes transiently expressing a hemagglutinin (HA)-tagged Snail protein (Figure 1B). The antibody also recognized a band corresponding to the size of endogenous Snail (approximately 28 kDa) in lysates from embryonic mouse skin, the temporal appearance of which corresponded to the waves of hair follicle morphogenesis from E15.5 to newborn when over 90% of the hair on the mouse is formed (Figure 1B). Consistent with the Western blot data, immunohistochemical analysis did not detect Snail in single-layered E13.5 epidermis (Figure 1C) nor in the placode, which is the earliest morphological sign of the commitment of multipotent cells of the embryonic ectoderm to a hair cell fate (Figure 1D). Consistent with the in situ hybridization results, anti-Snail antibody labeled only hair buds and not follicles at more mature stages of development (Figure 1E). Taken together, the anti-Snail antibody appeared to be specific for its target protein. It did not detect other Snail family members known to be expressed in keratinocytes and/or skin (unpublished data). Furthermore, the immunohistochemical data paralleled our *Snail* in situ hybridization data revealing transient *Snail* expression at the hair bud stage (Figure 1A).

As judged by immunohistochemistry, Snail protein was localized to the nuclei of the hair bud cells (Figure 1E). This feature was consistent with Snail's known function as a transcriptional repressor [12,13]. Additionally, anti-Snail labeling was detected in only three of the four major waves of follicle morphogenesis. Snail was not found in the buds of guard hairs that are the earliest of all hairs to form (at E13.5), and which constitute less than 5% of the mouse coat (unpublished data).

As judged by immunofluorescence with antibodies against the proliferating nuclear antigen Ki67, the timing of Snail expression coincided with the stage at which the developing follicle enhanced its proliferation and down-growth (Figure 1F). Immunohistochemistry with antibodies against the active (phosphorylated) form of MAPK (pMAPK) marked a subset of the proliferating (Ki67-positive) cells, and pMAPK-positive cells were enriched in the hair bud (Figure 1G). The timing of Snail induction and Ki67 and pMAPK enrichment in the hair bud appeared to follow closely the induction of LEF-1/β-catenin activity, known to initiate in the hair placode stage [20]. However, like placodes, hair buds exhibited down-regulation in E-cadherin expression (Figure 1H; see also [4]).

### Sustained Expression of Snail Results in Epidermal Hyperproliferation and Differentiation Defects in Tg Mouse Skin

The striking spike of Snail expression coincident with hair bud formation and enhanced proliferation prompted us to examine the consequences of ectopically expressing Snail elsewhere in mouse skin epidermis. To distinguish Tg from endogenous Snail, we used the HA-epitope, shown previously not to alter Snail's transcriptional activity [12]. Of 20 *K14-Snail[HA]* Tg animals generated, three expressed the transgene and all exhibited analogous phenotypes. Mice that integrated the transgene at the single-cell stage died at or shortly after birth. The three surviving full-Tg founder mice harbored transgene integrations that gave stable transmission of mosaic *Snail* gene expression through the germline. Progressively poor health necessitated our sacrificing most offspring from these lines within a year of birth.

**Figure 2:**
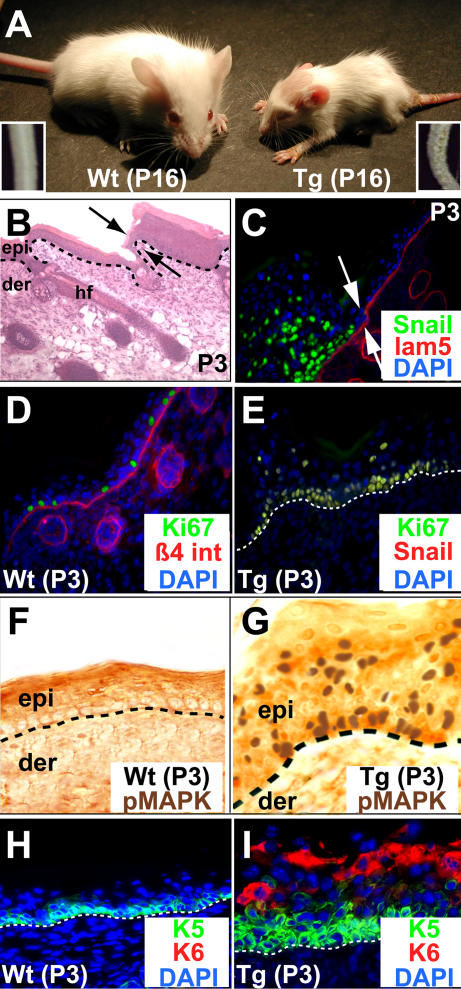
Misexpression of Snail in Mouse Skin Epidermis Results in Hyperproliferation Three different surviving Tg founder mice harbored a *K14-Snail* transgene that was integrated into a locus that resulted in inheritable, mosaic expression of the transgene in skin epidermis. All displayed similar abnormalities, as did their offspring. (A) P16 WT and *K14-Snail* Tg mice. Insets denote magnified tail segments, which displayed a mosaic, flaky appearance in Tg mice. Size differences appeared with age, and are likely due to K14-promoter activity in the tongue and oral epithelium, resulting in progressive defects and reduced food intake. Hence, skin sections from young (P3) mice were analyzed (B–I). (B) Hematoxylin- and eosin-stained Tg skin section. Double arrows demarcate the border of mosaic histology, with seemingly normal epidermis (epi) and a mature hair follicle (hf) at left and hyperthickened epidermis at right. (C) Immunofluorescence of Tg skin section labeled with antibodies as color-coded on frame. Double arrows demarcate the border of mosaic anti-Snail (green), revealing Snail expression coincident with regions of hyperthickened epidermis (at left) and absent in regions of normal epidermis (at right). (D–I) Sections of P3 WT or Tg skin (affected region) subjected to either immunofluorescence (D, E, H, and I) or immunohistochemistry (F and G) with antibodies as indicated on the panel. Anti-keratin 5 indicates K5, normally restricted to the basal layer of the epidermis; anti-keratin 6 detects keratin 6, expressed in postnatal epidermis under conditions such as wounding, in which hyperproliferation occurs. All other antibodies are as in the legend to Figure 2. Comparison of D and E provide representative examples that illustrate that pMAPK is found in only a subset of all proliferating (Ki67-positive) cells. Note also the presence of Ki67- (E) and pMAPK-positive (G) cells in some suprabasal areas; Ki67-positive cells colabeled with anti-Snail (E).

As *Snail* Tg animals grew, they became distinguished by their small size, short tails, and flaky skin (Figure 2A). Histological analyses of 3-d old (P3) mice revealed mosaic patches marked by epidermal thickening (Figure 2B). The mosaic morphology was reflected at the level of Tg Snail protein, with only the hyperthickened regions expressing nuclear HA-tagged Snail (Figure 2C). These hyperthickened areas were marked by excessive proliferation, as revealed by antibodies against the proliferating nuclear antigen Ki67 (Figure 2D and 2E). Activated, pMAPK-positive cells were also prevalent in these areas (Figure 2F and 2G), as were cells expressing keratin 6, a keratin induced in the suprabasal layers of hyperproliferative skin (Figure 2H and 2I).

**Figure 3:**
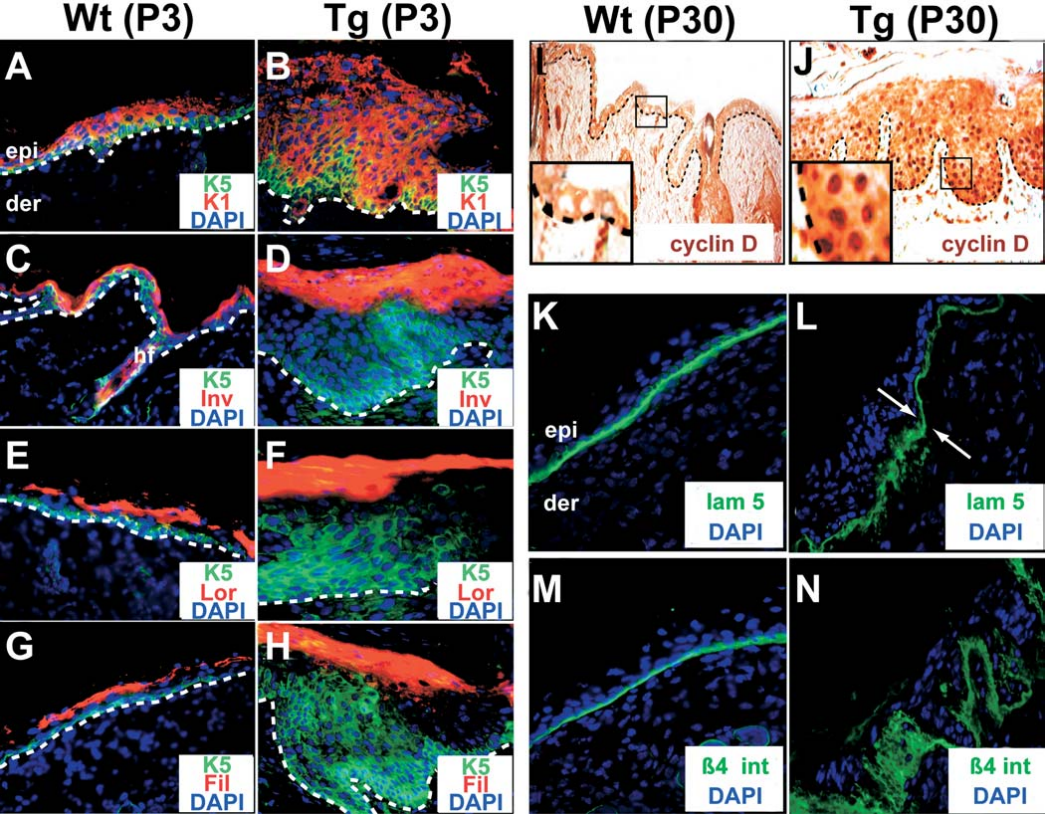
Alterations in the Differentiation Program and Basement Membrane Organization in Snail-Expressing Tg Epidermis (A–H) Immunofluorescence of skin sections from P3 WT and Tg mice. Shown are affected areas of Tg skin; in areas where Snail protein was not expressed, stainings were normal. Sections were labeled with antibodies as indicated and color-coded on each frame. Antibodies are against markers of normal epidermal differentiation, and include K5 (a basally expressed keratin), K1 (a suprabasal keratin, expressed in spinous layer cells), involucrin (Inv; a suprabasally expressed cornified envelope protein found in upper spinous and granular layer cells), loricrin (Lor; a cornified envelope protein expressed in the granular layer), and filaggrin (Fil; a protein that bundles keratin filaments in the granular layer and stratum corneum). Note abnormal extension of anti-K5 suprabasally, often present in anti-K1 positive suprabasal Tg cells. (I–N) Immunohistochemistry (I and J) or immunofluorescence (K–N) of sections of P30 Wt (I, K, and M) and Tg (J, L, and N) (affected areas) skins using the antibodies indicated. Note that with age, affected areas of the Tg epidermis became increasingly undulating, often exhibiting papilloma-like invaginations (J). Insets in I and J are magnified views of the boxed areas, illustrating the absence (Wt) or presence (Tg) of nuclear anti-cyclin D staining. With age, affected areas of the Tg epidermis also displayed perturbations within the basement membrane, as judged by antibody labeling against either basement membrane (K and L) or hemidesmosomal (M and N) components. Double arrows in L demarcate mosaic zones, revealing that perturbations were restricted to hyperthickened, i.e., Snail-positive zones (to left of double arrows). Other abbreviations are as noted in the legend to Figure 2.

Expression of the *Snail* transgene did not block terminal differentiation in the hyperproliferative epidermis, but it distorted it markedly (Figure 3A–3H). Typical of most hyperproliferating conditions, Snail expression led to a large expansion in layers with spinous and granular morphology. Additionally, however, was a marked and variable expansion of keratin 5 (K5), normally restricted to the innermost basal layer (see Figure 3). Although the failure of *Snail*-null mice to develop past gastrulation [21] precluded our ability to study the loss of Snail function in skin development, a good correlation emerged between the expression of Snail protein and the extension of K5, Ki67, and pMAPK suprabasally (compare data in Figures 2 and 3).

The changes in hyperproliferation and differentiation were not initially accompanied by gross signs of epithelial invaginations. With age, however, epidermal folds and undulations developed in areas where Snail was expressed, and proliferative markers persisted in these regions (Figure 3I and 3J; anti-cyclin D staining). The undulations were accompanied by partial dissolution of the underlying basement membrane (Figure 3K and 3L). Aberrant staining was also observed with antibodies against components of the hemidesmosomes, which provide strong adhesion of basal epidermal cells to the underlying basal lamina (Figure 3M and 3N). Interestingly, similar types of alterations occur in the basement membrane in the hair bud of embryonic and newborn mice when Snail is normally expressed. The fact that the basement membrane separating the epidermis from the dermis is altered only in the adult Tg animals suggests the involvement of intermediary factors not as readily available in the epidermis as they are in the follicle.

### Possible Links between Epidermal Hyperproliferation and Down-regulation of AJ Proteins in *Snail* Tg Mice

**Figure 4:**
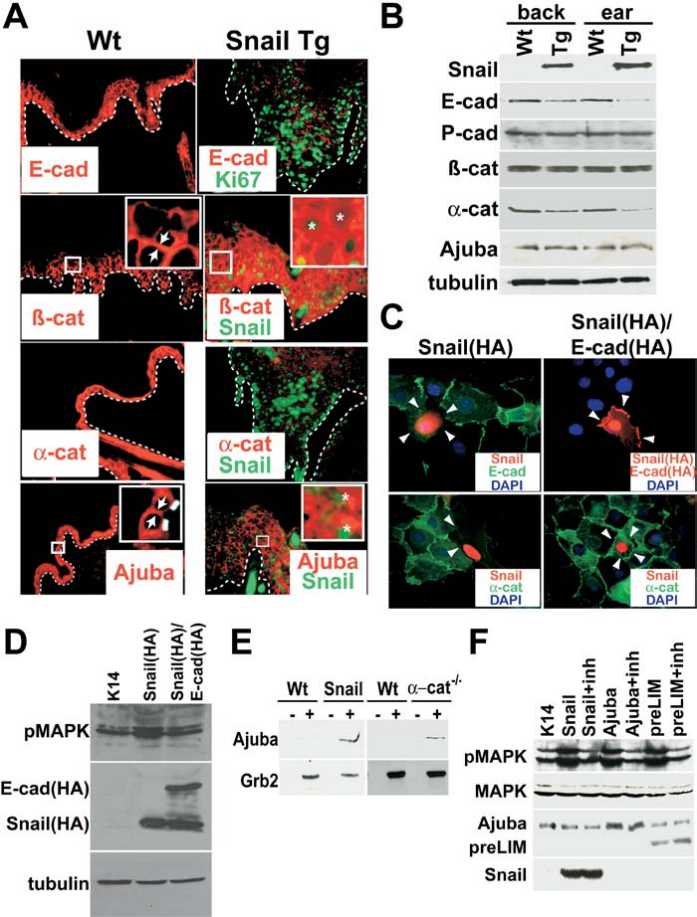
Snail-Mediated Remodeling of AJs Contributes to Hyperproliferation (A) Immunofluorescence of skin sections from P30 Wt and Tg mice. Shown are affected areas of Tg skin; in areas where Snail protein was not expressed, stainings were normal. Antibodies used are against AJ proteins and include E-cadherin (E-cad), the transmembrane core protein; β-catenin (β-cat), which binds E-cadherin at AJs and which can also participate as a transcription cofactor when associated with LEF-1/TCF proteins in the nucleus; α-catenin (α-cat) which binds to both β-catenin and Ajuba, a close relative of zyxin; and Ajuba, which can associate with proteins that bind to the actin cytoskeleton, as well as with Grb-2, a mediator of the GTP nucleotide-exchange protein Sos, involved in activation of the Ras-MAPK signaling cascade. In Snail-expressing Tg regions, there was a reduced staining with anti-E-cad and anti-α-cat and a more diffuse staining with anti-Ajuba. Insets in the panels for β-catenin and Ajuba staining are magnified views of the boxed areas. Arrows mark membrane localization of the protein and asterisks mark cells with elevated levels of cytoplasmic β-catenin or Ajuba. (B) Western blot analyses of protein extracts from P30 Wt and Tg back and ear skins. Antibodies are as in (A) except anti-P-cad, which detects P-cadherin, whose expression in the hair follicle was not affected, and anti-tubulin, which detects tubulin, a control for equal protein loadings. Note that the reductions seen in E-cadherin and α-catenin are likely to be underestimates of the actual differences in affected regions, since the Tg skin expressed Snail mosaically. (C) In the presence of elevated Snail, α-catenin levels can be restored by overexpression of E-cadherin. Keratinocytes were transfected with either HA-tagged Snail (Snail[HA]; images on the left) or Snail(HA) and Ecad(HA) (images on the right). 2 d after transfection, cells were switched from low-calcium growth medium to high-calcium medium for 6 h to induce AJ formation. Cells were stained with antibodies as indicated on the panels. Arrowheads point to sites of intercellular contact between a Snail-transfected keratinocyte and its neighboring untransfected cell. (D) Reintroduction *of E-cadherin* in keratinocytes expressing Snail returns pMAPK to basal levels. Keratinocytes were transfected with control vector (K14), or *Snail(HA),* or *Snail(HA)* + *E-cad(HA)*. After 2 d, cells were serum starved for 4 h and whole cell lysates were made and Western blotted with antibodies to pMAPK, HA to recognize the HA-tagged Snail and E-cadherin protein, 20or tubulin as a loading control. (E) Ajuba interacts with Grb-2 under conditions where α-catenin levels are reduced. Protein extracts were made from skins of P30 Wt and *K14-Snail* Tg P30 mice (blots on the left) and of newborn Wt and *K14-Cre/α-catenin (fl/fl)* conditionally null animals (blots on the right) [7]. Equal amounts of protein extracts were treated with anti-Grb-2 antibody (+) or control isotype antibody (–), and following centrifugation, immunoprecipitates were subjected to SDS-PAGE and Western blot analysis with anti-Ajuba and anti-Grb-2 antibodies. Note the presence of Ajuba only under conditions where levels of α-catenin and other AJ proteins were aberrantly low or absent. (F) Transgene expression of excess Ajuba or the Grb-2-interacting domain (pre-LIM) of Ajuba in keratinocytes results in the activation of the Ras-MAPK pathway. Primary newborn mouse keratinocytes were transfected with either the empty K14 expression vector (K14), or the expression vector driving Snail, full length Ajuba, or the pre-LIM domain of Ajuba in the absence or presence of a peptide inhibitor (inh) that disrupts the interaction between Grb-2 and Sos. 48 h posttransfection, protein extracts were prepared and subjected to SDS-PAGE and Western blot analyses with antibodies against pMAPK, total MAPK, Ajuba (also recognizing the smaller, pre-LIM domain), and Snail.

Given that the *E-cadherin* promoter is a direct target for Snail-mediated repression in vitro [4,12,13], and that E-cadherin was down-regulated in Snail-expressing hair buds, we examined the status of E-cadherin and other AJ proteins within regions of hyperproliferative epidermis where Tg Snail was present (Figure 4A). In these regions, immunofluorescence staining of E-cadherin and α-catenin were markedly diminished. In contrast, the intensity of antibody staining for two other AJ proteins, β-catenin and Ajuba, was still strong. Interestingly, however, despite appreciable immunofluorescence, localization of β-catenin and Ajuba appeared to be largely cytoplasmic rather than at cell-cell borders (Figure 4A insets).

Architectural differences in the epidermis made Western blot analyses somewhat difficult to gauge. However, in regions such as ear skin, where the highest levels of Snail protein were expressed, the effects were accentuated. In both back skin and ear skin, overall levels of E-cadherin and α-catenin were reduced, under conditions where β-catenin and Ajuba levels remained unchanged relative to controls (Figure 4B). Taken together, these data were consistent with our results obtained from immunofluorescence microscopy.

A priori, the decrease in α-catenin levels could be due to either direct transcriptional repression by Snail or perturbations in AJ formation caused by the decrease in *E-cadherin* gene expression. To distinguish between these possibilities, we tested whether α-catenin levels could be restored by exogenous expression of *E-cadherin* in Snail-expressing keratinocytes. As shown in Figure 4C, transiently transfected keratinocytes expressing HA-tagged Snail displayed a loss of E-cadherin and α-catenin at cell-cell borders. Coexpression of exogenous HA-tagged E-cadherin not only enabled cell-cell border localization of E-cadherin protein, but also rescued the cell-cell border staining of α-catenin (Figure 4C). The ability to restore α-catenin expression and localization under these conditions argues against the notion that Snail transcriptionally represses *α-catenin.* Rather, the findings are consistent with a previous report that E-cadherin is required for the translation of *α-catenin* mRNA [22].

Despite the reductions in AJ markers, Tg skin still displayed sealed membranes and intercellular junctions that were largely intact, as judged by ultrastructural analyses (unpublished data). In this respect, the skin epithelium resembled that of the hair bud, where the down-regulation in junction proteins is permissive for cell-cell remodeling without abrogating intercellular adhesion.

The similarities between *Snail* Tg epidermis and hair buds extended to the hyperproliferative state, leading us to wonder whether the down-regulation of AJ proteins might contribute to this condition. Given the increase in pMAPK staining in *Snail* Tg epidermis (see Figure 2G), we used pMAPK levels as our assay to test whether the loss of E-cadherin contributed to the Snail-mediated increase in proliferation. Consistent with our in vivo observations, transfected keratinocytes expressing Snail exhibited a substantial increase in pMAPK levels relative to control cells (Figure 4D). Coexpression of E-cadherin with Snail appeared to abrogate this effect. Together, these findings raised the possibility that an AJ-associated protein that is normally sequestered at the plasma membrane may participate in a proliferation signaling pathway when AJs are deconstructed.

Numerous studies have correlated a down-regulation of E-cadherin with a translocation of β-catenin to the nucleus and a transactivation of genes that are regulated by the LEF-1/T cell factor (TCF) family of DNA binding proteins [23,24,25]. The presence of nuclear cyclin D in hyperproliferative *Snail* Tg epidermis was particularly intriguing since prior studies have reported *cyclin D* gene as a direct target of TCF/β-catenin transcription [26]. This said, we did not detect nuclear β-catenin in our Tg epidermis, and mating the Snail Tg mice against the TOPGal reporter mouse [20] gave no signs of ectopic LEF-1/Tcf/β-catenin activity (unpublished data).

We next turned to the presence of cytoplasmic Ajuba for a possible mechanistic link to the proliferative increase in our *Snail* Tg epidermis. In addition to its documented ability to bind α-catenin [10], Ajuba can also associate with growth factor receptor-bound protein-2 (Grb-2)/son of sevenless (Sos), the nucleotide exchange factor for Ras, which is upstream from activation of MAPK [9]. Given the increase in pMAPK staining in Tg skin, we examined the possibility that Ajuba might have changed its binding partner in Snail-expressing epidermis. Interestingly, Ajuba was readily detected in anti-Grb-2 immunoprecipitates of protein lysates from skins of *Snail* Tg mice but not from the corresponding wild-type (WT) animals (Figure 4E). When these experiments were repeated with *α-catenin*-null epidermis, a similar Grb-2-Ajuba association was detected, and again, this interaction was not detected in the protein extracts from control littermate skin (Figure 4E). Together, these data demonstrate that the reduction in α-catenin levels, either by Snail-mediated down-regulation of *E-cadherin* or by *α-catenin* conditional targeting, allows Ajuba to interact with Grb-2/Sos.

If the competition between Grb-2/Sos and α-catenin for Ajuba is functionally relevant to the hyperproliferative state of a keratinocyte, then overexpression of Ajuba would be expected to bypass the competition and promote activation of the Ras-MAPK pathway in WT keratinocytes. Indeed, when serum-starved keratinocytes were transiently transfected with an Ajuba expression vector, the levels of pMAPK were not only elevated but also comparable to those transfected with the *K14-HASnail* transgene (Figure 4F). This activation was abolished when cells were treated with a small peptide inhibitor that specifically interrupts the Grb-2/Sos interaction (Figure 4F; see lanes marked “inh”) [27].

Ajuba's pre-LIM domain is the segment that associates with Grb-2's Src-homology 3 domain [9]. When this domain was overexpressed in serum-starved keratinocytes, a comparable elevation in pMAPK was observed (Figure 4F). As expected, the small peptide inhibitor that interrupts the Grb-2/Sos association blocked the effects. These data suggested that by elevating cytosolic Ajuba levels, Ajuba's pre-LIM domain may associate with Grb-2/Sos in a manner that stimulates its nucleotide exchange activity and leads to activation of the Ras-MAPK pathway. Although this pathway provides one mechanism by which Snail expression and proliferation may be coupled in skin epithelium, proliferative circuitries involving AJs are known to be complex and often interwoven. Future studies will be needed to systematically dissect these putative intricacies at a molecular level.

### Probing the Regulation of *Snail* Gene Expression Reveals an Essential Link to TGF-β2 Signaling in the Developing Hair Bud

The temporal spike of *Snail* mRNA expression in the hair bud prompted us to consider what factor(s) may be regulating the *Snail* gene. A variety of extracellular signals have an impact on the cell type-specific expression of different Snail family members, and many of them, including Wnts, BMPs, FGFs, and TGF-βs, also affect hair bud development [2,16,28]. Since Snail is not expressed in cultured skin keratinocytes that secrete active BMPs and FGFs (see Figure 1B), we focused our attention on Wnt and TGF-β signaling as more likely candidates for Snail induction in this cell type.

**Figure 5:**
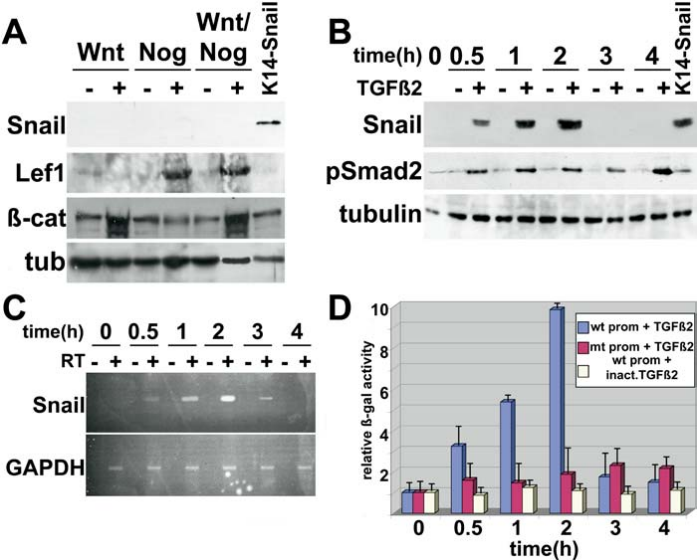
TGF-β2, but Not Wnt/noggin, Transiently Induces Snail Expression In Vitro (A) Failure of Wnt and noggin signaling to induce Snail in cultured keratinocytes. Primary mouse keratinocytes were treated with Wnt- and/or noggin-conditioned medium (+) or the corresponding control medium (–). These conditions are known to activate the LEF-1/β-catenin reporter TOPGal and down-regulate the *E-cadherin* promoter (see [4] for details). Using Western blot analyses, cellular proteins were then analyzed for Snail, LEF-1, β-catenin, and tubulin. Proteins from keratinocytes transfected with K14-*Snail* were used as a positive control for Snail expression. (B) TGF-β2 can induce Snail protein. Primary keratinocytes were treated for the indicated times with recombinant TGF-β2 (+) or heat inactivated TGF-β2 (–).Total cellular proteins were then isolated and analyzed by Western blot for Snail, pSMAD2 (reflective of activated TGF- signaling), and tubulin. Note the activation of Snail expression, peaking at 2 h post-TGF-β2 treatment and then disappearing thereafter. (C) *Snail* mRNA expression is transiently induced by TGF-β2. The experiment in (B) was repeated, and this time, total RNAs were isolated from keratinocytes treated with TGF-β2 for the indicated times. RT-PCR was then used with (+) or without (–) reverse transcriptase (RT) and with primer sets specific for *Snail* and GAPDH mRNAs. Note that *Snail* mRNA expression also peaked at 2 h, paralleling Snail protein. (D) TGF-β2 treatment results in enhanced activity of a *Snail* promoter-β-galactosidase reporter. Keratinocytes were transfected with a β-galactosidase reporter driven by a *Snail* promoter that is either WT (wt prom) or harbors a mutation in a putative pSMAD2/pSMAD4 binding site (mt prom). At 2 d posttransfection, cells were treated with either TGF-β or heat-inactivated TGF-β2 (inact) for the times indicated. β-galactosidase assays were then conducted, and results are reported as fold increase over a basal level of activity of 1. The experiment was repeated three times in triplicate, and error bars reflect variations in the results.

Previously, we showed that effective transmission of a Wnt-3a signal in cultured keratinocytes can be achieved through their exposure to the BMP inhibitor noggin, which induces LEF-1 expression [4]. In vitro, these conditions down-regulated the *E-cadherin* promoter and induced a LEF-1/β-catenin-sensitive reporter gene, *TOPFLASH* [4]. In contrast, Snail expression was not induced by these conditions (Figure 5A). Thus, despite essential roles for Wnts and noggin in hair follicle specification [4,29,30], our studies did not support an essential role for these signals in governing *Snail* expression in keratinocytes.

TGF-β1 has been shown to induce Snail family members in hepatocytes and heart [15, 31]. In keratinocytes, however, TGF-β1 inhibits keratinocyte growth and seems to be involved in triggering the destructive phase of the cycling hair follicle [32]. Of the loss-of-function mutations generated in each of the *TGF-β* genes, only the *TGF-β2* null state blocked follicle development at the hair bud stage [32]. Thus, we turned towards addressing whether TGF-β2 might be involved in regulating *Snail* expression in keratinocytes isolated from the basal layer of the epidermis. Though there is no cell culture system available to specifically study placodal cells, these keratinocytes are their progenitors and are the closest approximation available to study the behavior of epithelial cells of the placode.

Interestingly, treatment of cultured keratinocytes with as little as 5 ng/ml of TGF-β2 caused a rapid and transient induction of Snail (Figure 5B). Following this treatment, Snail protein was detected within 30 min, peaked at 2 h, and then declined thereafter. The induction of Snail appeared to be specific for the active form of the growth factor, as pretreatment of TGF-β2 for 10 min at 100 °C obliterated the response [Figure 5B, lanes marked (–)]. By contrast, although TGF-β receptor activation remained elevated during the duration of the experiment (as measured by the sustained phosphorylation of the downstream effector SMAD2) *Snail* expression could not be maintained (Figure 5B). Thus, although *Snail* expression correlated with phosphorylated SMAD2 (pSMAD2) induction, its decline seemed to rely on secondary downstream events.

The rapid kinetics of Snail expression were reflected at the mRNA level, suggesting that *Snail* promoter activity in keratinocytes might be sensitive to TGF-β2 signaling (Figure 5C). To test this possibility, we engineered a transgene driving the β-galactosidase reporter under the control of approximately 2.2 kb of promoter sequence located 5′ from the transcription initiation site of the mouse *Snail* gene. At 2 d after transient transfection, keratinocytes were treated with TGF-β2 (t = 0) and then assayed for transgene activity over the same time course in which we had observed Snail protein induction. The results of this experiment are presented in Figure 5D.

Within 0.5 h of TGF-β2 treatment, *Snail* promoter activity had increased 3-fold, and by 2 h, it peaked to approximately 10-fold over control levels (Figure 5D). Thereafter, *Snail* promoter activity rapidly returned to the basal levels seen in unstimulated keratinocytes. The kinetics of *Snail* promoter activity closely paralleled those observed for Snail protein induction. Moreover, the stimulatory effects appeared to be specific to TGF-β2, since they were abrogated either by heat inactivation of the TGF-β2 protein or by mutation of a putative SMAD binding element located about 1.8 kb 5′ from the *Snail* transcription start site (Figure 5D). Taken together, these results suggested that in keratinocytes, TGF-β2 signaling results in a pSMAD2-dependent transient activation of the *Snail* gene, and that maintenance of Snail protein relies, in part, upon sustained promoter activity.

**Figure 6:**
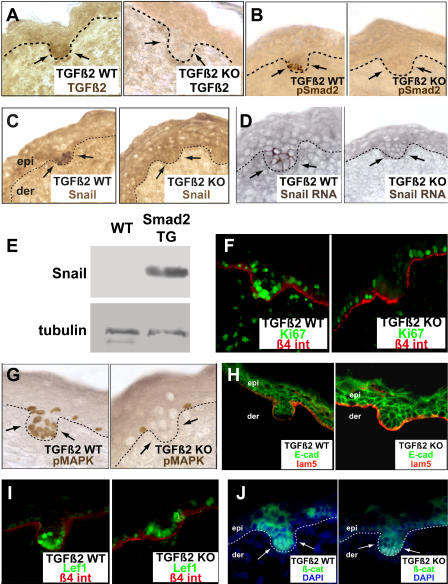
TGF-β2 Is Necessary to Induce Snail Expression and Regulate Proliferation and E-Cadherin in the Hair Bud (A–D) Skins from TGF-β2 WT or KO E17.5 embryos were analyzed for expression of TGF-β2 protein (A), which is present in the epidermis and dermis as previously described [33] and in the hair bud, pSMAD2 (B), Snail (C), and *Snail* mRNA (D). Arrows point to the hair buds. (E) Western blot analyses of Snail expression in the skins of 2-wk-old *K14-Smad2* transgenic (SMAD2 TG) and WT littermate (WT) mice. Antibody to tubulin was used as a control for equal protein loadings. The K14-Smad2 Tg mouse was previously shown to possess activated TGF-β signaling [35]. (F–G) Proliferation markers Ki67 (F) and pMAPK (G) are diminished in *TGF-β2*-null hair relative to its WT counterpart. (H–J) *TGF-β2*-null hair fails to down-regulate E-cadherin (H). Wnt and noggin signaling pathways are still intact in the TGF-β2 null hair as nuclear LEF-1 (I) and nuclear β-catenin (J) are still expressed.

The brevity of *Snail* gene and protein induction in TGF-β2 treated cultured keratinocytes resembled the temporal appearance of *Snail* mRNA and protein at the initiation of hair follicle morphogenesis in embryonic mouse skin. To test whether TGF-β2 might be required for *Snail* induction in hair bud formation in vivo, we first analyzed whether TGF-β2 was expressed in or around the hair bud. Consistent with previous observations [33], an anti-TGF-β2 antibody labeled developing hair buds (Figure 6A). This labeling appeared to be specific as judged by the lack of staining in follicle buds from mice homozygous for a *TGF-β2* null mutation (Figure 6A; [34]). Moreover, the downstream effector of TGF-β2 signaling, pSMAD2, was also expressed in WT, but not *TGF-β2*-null, hair buds (Figure 6B). Together, these data underscore the importance of the TGF-β2 isoform despite expression of both TGF-β1 and TGF-β2 in developing hair buds at this stage.

To further explore the possible relation between Snail and TGF-β2, we examined the status of Snail expression in *TGF-β2*-null hair buds. As judged by immunohistochemistry, Snail protein was absent from E17.5 skin of *TGF-β2-*null embryos but not from that of control littermates (Figure 6C). This effect appeared to be exerted at the transcriptional level, since *Snail* mRNAs were also not found in *TGF-β2* null hair buds under conditions in which the signal was readily detected in the hair buds of littermate skin (Figure 6D).

Conversely, in 2-wk-old *K14-Smad2* Tg mice, which display elevated TGF-β signaling in skin [35], Snail protein was readily detected by Western blot analyses, where it was not found in postnatal skin (Figure 6E). Taken together, these results provide compelling evidence that TGF-β2 is functionally important for inducing *Snail* gene expression in a pSMAD-dependent manner in developing hair buds. Whether pMARK activity also contributes to Snail induction was not addressed in the present study [15].

Although some hair buds still formed in *TGF-β2* null skin, their number was reduced by approximately 50% [32]. Thus, although the pathway mediated by TGF-β2 signaling impacts the earliest step of epithelial invagination, it does not appear to be essential for bud morphogenesis. Consistent with this notion, basement membrane remodeling still took place in the *TGF-β2*-null buds, as judged by immunofluorescence with antibodies against β4 integrin, an integral component of keratinocyte-mediated adhesion to its underlying basement membrane (Figure 6F). In contrast, TGF-β2 signaling appeared to be an important factor for the early proliferation that occurs in the developing hair buds, as judged by anti-Ki67 and anti-pMAPK immunofluorescence (Figure 6F and 6G).

If TGF-β2 stimulates *Snail* expression in developing buds, loss of this morphogen would be expected to affect the expression of genes that are typically repressed by Snail. Since a major target for Snail-mediated repression is the *E-cadherin* gene [12,13], we investigated the status of E-cadherin in *TGF-β2*-null buds. As shown in Figure 6H, hair buds in TGF-β2 null skin displayed elevated immunofluorescence staining relative to their WT counterparts.

Previously we demonstrated that the concerted action of the extracellular signals Wnt and noggin are required for the generation of a LEF-1/β-catenin transcription complex to repress *E-cadherin* transcription at the onset of hair fate specification. As shown in Figure 6I and 6J, both WT and TGF-β2 null buds exhibited nuclear LEF-1 and β-catenin localization, signs that the Wnt-noggin signaling pathway was intact. These data suggest that during hair follicle morphogenesis, TGF-β2 functions subsequently to Wnt/noggin-mediated determination of hair fate. Moreover, through activation of *Snail* gene expression, TGF-β2 appears to work in tandem with these other morphogens to down-regulate E-cadherin levels, which contributes to the activation of proliferative circuitries.

## Discussion

During budding morphogenesis, intersecting signaling networks from the epithelium and mesenchyme govern transcriptional, adhesive, polarity, and motility programs in these select groups of cells. The dynamic nuclear and cytosolic changes that take place during this time form the cornerstone for organ morphogenesis. Two major challenges in understanding the mechanisms underlying a particular budding process are to order the temporal sequence of external cues involved and then to dissect how the cells of the developing bud translate these signals into the downstream events of cellular remodeling, proliferation, and differentiation. Our studies here provide some insights into how these events are orchestrated during hair bud formation in developing skin.

### Signaling during Early Hair Follicle Morphogenesis

Recent studies on hair bud morphogenesis suggest that Wnt signals likely from the epithelium and BMP inhibitory signals from the underlying mesenchyme converge to produce an active transcription factor complex involving β-catenin and LEF-1, which in turn plays a key role in specifying the hair follicle fate [4,29,30,36,37]. Sonic hedgehog (Shh) and TGF-β2 signaling also play essential roles in follicle morphogenesis, but in contrast to *β-catenin* null skin, in which follicle invaginations are absent [30], some hair buds still form in the absence of LEF-1, Shh, or TGF-β2 [32,38]. These likely reflect the first wave of follicle (i.e., guard hair) morphogenesis, which accounts for a small number (fewer than 5%) of hairs and is under distinct regulatory control. Guard hairs form in the absence of LEF-1 and TGF-β2, and we have found that they also fail to express Snail at the budding stage of development (unpublished data). How E-cadherin is regulated in guard hairs remains to be determined. Several candidates include other Snail family members such as Slug or twist, or alternatively, transcription factors involving β-catenin and a different member of the LEF-1/TCF/*Sry*-type HMG box (commonly known as SOX) family [39,40]. Further investigation will be required to determine whether the signaling pathway we have elucidated here is a theme with multiple variations.

TGF-βs are known to promote withdrawal of keratinocytes from the cell cycle [41]. Hence, when TGF-β2 protein was detected at the transition between the growing and destructive phases of the adult hair cycle, research initially and naturally focused on a role for this family member in cessation of growth and/or triggering apoptosis ([42] and references therein). However, in contrast to *TGF-β1*-null skin, which exhibits an extended growing phase of postnatal hair follicles, *TGF-β2*-null skin displays an embryonic block in follicle bud progression [32]. Although this phenotype is consistent with TGF-β2's embryonic expression patterns [33], about 50% of *TGF-β2* null buds appear unable to progress to the down-growth phase, a feature that cannot be explained readily on the basis of previously established effects of TGF-βs.

Our finding that TGF-β2 is upstream from Ki67 expression and MAPK activation lends further support to the notion that hair follicle keratinocytes at this early stage of development react to TGF-β2 signaling in a fashion opposite to that typically expected for TGF-β factors. This said, based upon pSMAD2 immunohistochemistry, the immediate steps of downstream signaling appeared to be intact. Thus, we surmise that the proliferative outcome is likely to be rooted in differences in the repertoire of activated SMAD target genes. In this regard, the positive effects of TGF-β2 on proliferation within the hair bud may be more analogous to what has been seen in progression of squamous cell carcinoma to metastatic carcinoma [43] rather than that typically observed for keratinocytes [44,45,46].

The prior identification of the *Snail* gene as a potential target of TGF-β signaling [15] was intriguing, given the temporal wave of *Snail* gene expression that occurs in the developing hair bud. The additional correlation between epithelial hyperproliferation and *Snail* transgene expression further fostered our interest in a possible link between TGF-β2 and Snail. Our functional studies demonstrate that without TGF-β2, *Snail* expression is abolished in the mutant hair buds, and conversely, in *K14-Smad2* skin, *Snail* is ectopically activated. Moreover, our in vitro studies indicate that even sustained TGF-β2 exposure may cause only a transient induction of Snail, offering a possible explanation as to why Snail is so briefly expressed during hair follicle morphogenesis. An additional point worth mentioning is that prolonged expression of Tg *Snail* in postnatal skin resulted in morphological and biochemical signs of epithelial to mesenchymal transitions (unpublished data), underscoring why transient Snail expression may be so important during normal hair follicle morphogenesis [18].

At first glance, the sparsity in hair coat of K14-*Snail* Tg mice seemed indicative of a defect in follicle formation (see Figure 2A). Closer inspection, however, revealed that not all hairs penetrated the hyperthickened Tg epidermis. Several factors may contribute to the seemingly normal follicle development in these mice. One obvious factor is the K14 promoter, which is elevated in the basal layer of the epidermis and the outer root sheath (ORS) of the hair follicle, but is markedly down-regulated in developing embryonic hair buds as well as in the postnatal hair progenitor cells. The K14 promoter is also less active in the ORS than epidermis and hence this might also account for the lack of apparent response of the ORS to ectopic Snail. Additional contributing factors could be the multiplicity of Snail family members and their differential expression, the saturation, and/or diversity of regulatory mechanisms that govern AJ formation, migration, and proliferation in the follicle ORS. Distinguishing between these possibilities must await the generation of mice harboring skin-specific loss-of-function *Snail* mutations.

### Links between Signaling, Transcriptional Cascades, Epithelial Remodeling, and Proliferation in the Hair Bud

Previously, we discovered that early during hair follicle morphogenesis, *E-cadherin* gene expression is down-regulated concomitantly with the invagination of developing bud cells into the skin [4]. Because the timing of this event correlated with the activation of a LEF-1/β-catenin transcription factor complex [20], we were intrigued by the presence of a putative LEF-1/TCF binding site in the *E-cadherin* promoter. This prompted an investigation that subsequently led to our discovery that LEF-1/β-catenin can contribute to repression of *E-cadherin* gene expression in skin keratinocytes [4]. In the course of these studies, we also noted that Snail can also contribute to this process in keratinocytes in vitro, and our present studies revealed that Snail is expressed at the right place and time to be physiologically relevant in the process.

In *noggin*-null embryonic skin, LEF-1 expression and subsequent activation of the LEF-1/β-catenin reporter gene is abrogated in the developing placodes. The corresponding failure of *E-cadherin* down-regulation underscores the importance of Wnt/noggin signaling in regulating this event in follicle morphogenesis [4]. Conditional gene targeting studies will be necessary to establish whether Snail family members also contribute to the down-regulation in *E-cadherin* gene expression that occurs during follicle formation. However, it is intriguing that *K14-Snail* Tg epidermis displayed a marked down-regulation in E-cadherin expression, thereby demonstrating its potential to do so in skin. Our prior findings showed that by elevating E-cadherin levels or by conditionally ablating *α-catenin,* hair follicle morphogenesis can be impaired [4,7]. The marked epidermal hyperproliferation seen in the *K14-Snail* Tg skin, coupled with the converse suppression of proliferation and Snail in *TGF-β2*-null hair buds led us to wonder whether the down-regulation of E-cadherin during follicle morphogenesis might have a direct impact on elevating the proliferative state of these cells.

Our Tg studies suggested that, at least in part through its regulation of *E-cadherin*, Snail is able to influence the subcellular localization of a variety of AJ-associated proteins. One of these appears to be Ajuba, which was previously shown to have the dual capacity to bind Grb-2 as well as α-catenin [9,10]. Our studies revealed that in skin keratinocytes that either harbor a conditional null mutation in *α-catenin* or that overexpress Snail, Ajuba develops an interaction with Grb-2 that is otherwise not observed in WT keratinocytes. The corresponding abilities of either *Snail*-transfected or *Ajuba*-transfected keratinocytes to exhibit elevated activation of the Ras-MAPK pathway suggest that the Grb-2 association of Ajuba under conditions of reduced levels of AJ proteins may be directly relevant to the parallel in hyperproliferation.

In stable epithelial (i.e., Madin-Darby canine kidney, or MDCK) cell lines, Snail has been shown to block cell cycle progression and promote motility and shape changes for invasion [47]. While our in vivo studies are consistent with a role for Snail in motility and epithelial remodeling, they differ with respect to Snail's apparent proliferative effects. A priori, this could be simply due to variations in the response of different cell types to Snail expression. Alternatively, these differences may be relevant to the benefit of using mouse models to reveal functions not always recapitulated in stable cell line models. Future studies should highlight the underlying reasons for these opposing results.

Irrespective of these differences, our in vivo studies do not stand alone, as there are many situations in which a down-regulation in AJ proteins correlate with enhanced proliferation. In fact, a myriad of diverse mechanisms have been implicated in activating epithelial proliferation upon down-regulation of AJ proteins [7,23,24,48]. Sifting through these converging pathways is likely to be a difficult and painstaking process. This said, by identifying the status of different players involved in specific cell types and at specific stages in development, our mechanistic understanding of how intercellular remodeling is linked to proliferation in epithelial morphogenesis should begin to surface in the future. Elucidating the molecular mechanisms through which these networks converge is also a prerequisite for understanding how these processes go awry during tumorigenesis.

## Materials and Methods

### 

#### Reagents

Primary antibodies used were against: E-cadherin (M. Takeichi, Kyoto University, Japan); α-catenin, β-catenin, pMAPK, tubulin (Sigma, St. Louis, Missouri, United States), Ajuba (G. Longmore, Washington University, St. Louis, Missouri, United States); β4 integrin/CD104 (BD Pharmingen, San Diego, California, United States), laminin 5 (R. Burgeson, Harvard University, Cambridge, Massachusetts, United States), K5, K1, loricrin (Fuchs Lab), involucrin, fillagrin (Covance, Berkeley, California, United States), MAPK, pSMAD2 (Cell Signaling, Beverly, Massachusetts, United States); Grb-2 (Santa Cruz Biotech, Santa Cruz, California, United States); P-cadherin (Zymed Laboratories, South San Francisco, California, United States); HA (Roche Biochemicals), vimentin (Chemicon, Temecula, California, United States), Ki67 (Novo Castra, Newcastle Upon Tyne, United Kingdom), keratin 6 (P. Coulombe, John Hopkins University, Baltimore, Maryland, United States), cyclin D (Oncogene, San Diego, California, United States), and TGF-β2 (L. Gold, New York University, New York, New York, United States). FITC-, Texas Red-, or HRP-conjugated secondary antibodies were from Jackson ImmunoResearch (West Grove, Pennsylvania, United States). Biotinylated secondary antibodies were from Vector Labs (Burlingame, California, United States). Dilutions were according to the manufacturer's recommendation. The Snail antibody was generated in Guinea pigs by inoculating them with the N-terminal sequence of murine *Snail* fused to GST (Covance, Princeton, New Jersey, United States). Recombinant human TGF-β2 was purchased from R&D (Minneapolis, Minnesota, United States). Heat inactivated TGF-β2 was generated by heating the recombinant protein at 100 °C for 10 min.

#### Mice

The *K14-Snail* Tg mouse was generated by digesting the pcDNA3-mm *Snail*-HA plasmid (G. de Herreros, Universitat Pompeu, Fabra, Barcelona, Spain) with BamHI and NotI and subcloned into the K14 vector [49]. The linearized construct was injected into the nucleus of embryos from CD1 mice. The *K14-Smad 2* Tg mouse was reported in Ito et al., 2001. The *TGF-β2* knockout (KO) mouse was described in [34]. The *shh* KO mouse [38] and TOPGal mouse [20] have previously been reported.

#### Western blot and immunoprecipitation

Protein extracts from primary keratinocytes were generated either by lysing cells in lysis buffer (1% NP-40, 1% sodium deoxycholate, 20 mM Tris-Cl [pH 7.4], 140 mM NaCl containing 1 mM sodium vanadate, 2 mM phenylmethylsulfonyl fluoride, and protease inhibitors) or directly in Laemmli bβuffer and boiled. For skin tissue: Frozen tissue was pulverized in a liquid nitrogen-cooled Gevebesmascher and the powder scraped into a chilled microcentrifuge tube. RIPA buffer (1% Triton X-100 in PBS with 10 mM EDTA, 150 mN NaCl, 1% sodium deoxycholate, and 0.1% SDS) and protease inhibitors or Laemmli buffer was added. The cell suspension was sonicated three times for 15 s and centrifuged at 14,000 rpm at 4 °C. The supernatant was separated from the pellet and used in the experiments. Extracts subjected to immunoprecipitation were precleared with Protein G Sepharose (Amersham, Piscataway, New York, United States) and incubated with antibody with rocking overnight at 4 °C. Protein G Sepharose was added and samples were incubated for 1 h at 4 °C with rocking. Samples were washed three times for 5 min each in lysis buffer, and the Protein G Sepharose-antibody-antigen pellet was resuspended in Laemmli buffer and boiled for 10 min. Samples were run on SDS-PAGE and transferred to nitrocellulose membrane (Schleicher and Schuell Bioscience, Keene, New Hampshire, United States). Western blot signals were developed using the enhanced chemiluminescence kit from Amersham

#### Cell culture

Primary keratinocytes were culture in low-calcium medium as previously described [4]. Transient transfections were carried out with FuGENE6 reagent (Roche, Indianapolis, Indiana, United States) according to the manufacturer's protocol. Measurement of β-galactosidase or luciferase levels in promoter activity studies were carried out with the Galacto-Lite assay kit (TROPIX, Bedford, Massachusetts, United States) and the Dual luciferase (Promega, Madison, Wisconsin, United States), respectively. Runella luciferase was cotransfected into cells to correct for transfection efficiency. Experiments were done in triplicate and repeated at least three times. Measurements were done on a luminometer (MGM Instruments, Hamden, Connecticut, United States). For experiments measuring phosphorylation of MAPK, keratinocytes were serum starved for 3 h prior to harvesting of cells by incubation in medium lacking serum. Treatment of cells with Wnt- and noggin-conditioned medium was previously described [4].

#### Constructs

The 2.2-kb murine *Snail* promoter was generated by PCR using a forward primer with an XbaI linker sequence, 5′- TCTAGAATTGTTTGCTGCTGTATGGTCTTC-3′, along with a reverse primer with a BglII linker sequence, 5′- AGATCTGTTGGCCAGAGCGACCTAG- GTAG-3′, and mouse genomic DNA as a template. The PCR product was purified with the Gel Extraction Kit (Qiagen, Valencia, California, United States) and ligated into pCRII-TOPO TA vector (Invitrogen, Carlsbad, California, United States). The promoter was verified by sequencing and digested with XbaI and BglII and subcloned into the pβ-gal BASIC vector (BD Biosciences Clontech, Palo Alto, California, United States). The point mutations in the SMAD binding element was generated with the Quik-Change Kit (Stratagene, La Jolla, California, United States) using the forward primer 5′- GGGCGGGCTTAGGTGTTTTCATTTACTCTTGAGGAAAAGCTTGGC-3′ and the reverse primer 5′- GCTTTT- CCTCAAGAGTAAATGAAAACACCTAAGCCCGCCCTGCCC-3′. The probes for the *Snail* in situ hybridization were generated against the 3′ UTR by PCR using the forward primer 5′- ACCTTCTCCCGCATGTCCTTGCTCC-3′ and the reverse primer 5′- CTGCTGAGGCATGGTTACAGCTGG-3′, and genomic DNA as a template. The PCR product was gel purified and ligated into pCRII-TOPO TA vector. The pre-LIM domain of Ajuba was generated essentially as described [9], but was fused to GFP by subcloning from the pEGFP-N1 20 vector (BD Biosciences Clontech)

#### In situ hybridization

The pCRII-TOPO TA vector containing a region of the 3′ UTR of *Snail* was used as a template to generate digoxigenin-labeled sense and antisense riboprobes (Roche). The respective probes were obtained by XhoI and BamH1 digestions. In situ hybridizations were performed on 10-μm thick sections of E17.5 mouse embryos. The sections were fixed with 4% PFA for 10 min at room temperature, prehybridized at room temperature for 4.5 h, hybridized with the probe (2 μg/ml) at 55 °C for 12–14 h, blocked with 10% NGS, and treated with anti-dig Fab-AP antibody (Roche #1093274) at a 1:2,500 dilution for 3 h. The sections were incubated with NBT and BCIP until adequate signal was detected.

#### Immunofluorescence and immunohistochemistry

Tissue samples for immunofluorescence were frozen in OCT and sectioned 10 μm thick on a cryostat. Sections were fixed in 4% paraformaldehyde for 10 min at room temperature, blocked, and stained with antibodies. Tissue samples for immunohistochemistry were fixed in 4% paraformaldehyde, dehydrated, and embedded in paraffin. Samples were sectioned on a microtome (10 μm thick) and rehydrated prior to staining with antibody. Samples stained with Snail, pMAPK, pSmad2, and cyclin D were antigen unmasked with 10 mM sodium citrate (pH 6) in an Antigen Retriever 2100 (Pickcell Laboratories, Leiden, Netherlands). The DAB substrate kit (Vector Labs) was used according to manufacturer's instructions to develop the signal.

#### RT-PCR

RNA was extracted from keratinocytes or skin tissue with Trizol (Invitrogen) according to the manufacturer's protocol. cDNA was generated using oligo-dT primers and the Superscript II kit (Invitrogen). The primers used for PCR were *Snail* forward: 5′- CAGCTGGCCAGGCTCTCGGT-3′; *Snail* reverse: 5′- GCGAGGGCCTCCGGAGCA-3′; GAPDH forward 5′- CGTAGACAAAATGGTGAAGGTCGG-3′; and GAPDH reverse: 5′- AAGCAGTTGGTGGTGCAGGATG-3′.

## Abbreviations

AJ: adherens junction
BMP: bone morphogenic protein
EMT: epithelial to mesenchymal transition
E[number]: embryonic day [number]
FGF: fibroblast growth factor
Grb-2: growth factor receptor-bound protein-2
HA: hemaglutinin
K1: keratin 1
K5: keratin 5
KO: knockout
LEF-1: lymphoid enhancer factor-1
LIM: Lin-1
MAPK: mitogen-activated protein kinase
ORS: outer root sheath
pMAPK: phosphorylated MAPK
P[number]: postnatal day [number]
pSMAD: phosphorylated SMAD
Shh: sonic hedgehog
SMADs: small phenotype– and mothers against decapentaplegic–related proteins
Sos: son of sevenless
SOX: *Sry*-type HMG box
TCF: T cell factor
Tg: transgenic
TGF-β: transforming growth factor β
TOP: TCF-optimal-promoter
UTR: untranslated region
WT: wild-type
